# Comparative effectiveness of 7 major human let-7-5p isoforms to modulate target gene expression in liver cells

**DOI:** 10.1016/j.dmd.2026.100260

**Published:** 2026-03-02

**Authors:** Joseph M. Cronin, Mei-Juan Tu, Yimei Wang, Ai-Ming Yu

**Affiliations:** Department of Biochemistry and Molecular Medicine, University of California - Davis, School of Medicine, Sacramento, California

**Keywords:** MDR, ABC transporters, let-7 isoforms, Bioengineered RNAs, PTGR, HCC

## Abstract

Dysregulation of efflux ATP-binding cassette (ABC) transporters often confers multidrug resistance, presenting significant challenges in treating various diseases (eg, hepatocellular carcinoma [HCC]). The let-7-5p microRNAs (miRNAs), commonly downregulated in HCC, have established roles in controlling post-transcriptional gene regulation of ABC transporters (eg, multidrug resistance–associated protein 5 MRP5/ABCC5) and some oncogenes (eg, RNA-binding protein LIN28B). Although previous research has demonstrated the potential of particular let-7-5p isoforms to regulate ABC transporters and inhibit HCC cell viability, the comparative efficacy of let-7-5p isoforms whose sequences differ in several nucleosides is unknown. This study was to compare the effectiveness of 7 major let-7-5p isoforms (let-7a to let-7g) to regulate ABCC5 and LIN28B targets and inhibit HCC cell viability in vitro by using novel bioengineered RNA let-7-5p (BioRNA/let-7-5p) agents. Release of let-7-5p isoforms from individual BioRNA/let-7-5p molecules in Huh7, HepG2, and Hep3B cells was validated. Efficacy of BioRNA/let-7-5p isoforms to repress ABCC5/MRP5 and LIN28B protein levels was found to be target dependent; among them, let-7c and let-7d-5p exhibited broader regulatory efficacy against ABCC5/MRP5, while let-7d-5p emerged as the most potent suppressor of LIN28B, generally in accordance with let-7-5p abundance and target complementarity. By contrast, let-7-5p isoforms showed minimal impact on ABCC2/MRP2 and ABCC4/MRP4 protein levels. In addition, let-7-5p isoforms showed variable efficacy to inhibit the viability of different HCC cells. Together, our studies established the functional differences of let-7-5p isoforms in regulating target gene expression and inhibiting HCC cell viability, providing insights into intrinsic differences of miRNA isoforms to inform rational development of miRNA therapeutics or combination therapy.

**Significance Statement:**

Using novel bioengineered RNA agents, this study established the functional differences of 7 major human let-7-5p isoforms to control target gene expression and hepatocellular carcinoma cell viability in vitro. These findings demonstrate the potential of bioengineered RNA molecules to interrogate post-transcriptional gene regulation mechanisms, highlighting specific let-7-5p isoforms to modulate transporter and oncogene expression toward the development of improved therapies.

## Graphical abstract

**Figure:**
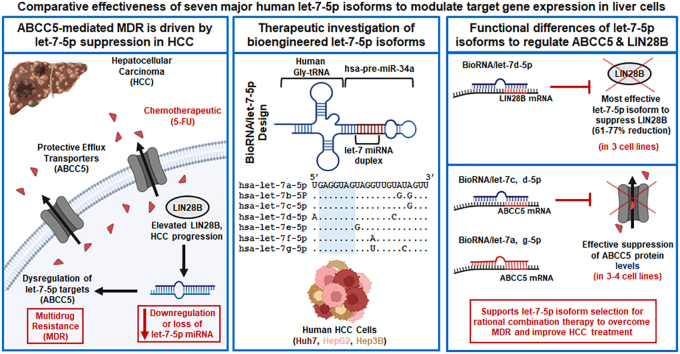

## 1 Introduction

Multidrug resistance (MDR) is a critical challenge in anticancer and antimicrobial therapies, hindering the successful treatment of hepatocellular carcinoma (HCC), the most prevalent form of liver cancer.^1^ In HCC, MDR is often driven by the overexpression of efflux ATP-binding cassette (ABC) transporters or multidrug resistance–associated proteins (MRP), including ABCB1/MDR1, ABCC1-5/MRP1-5, and ABCC10/MRP7.^2^ These transporters allow cancer cells to pump out many chemotherapeutic drugs, reducing intratumoral drug exposure and thus therapeutic efficacy.^3^, ^4^, ^5^ Overexpression of these transporters is a hallmark of chemo-resistant HCC and has been linked to poor survival and therapeutic evasion, posing a major challenge for current HCC therapies.^5^ Considering these challenges, creative interventions to overcome MDR are urgently needed for improving the treatment of HCC.

To develop effective therapies against resistant cancers, it is crucial to understand the underlying factors contributing to variable drug responses. Administered drugs are subject to absorption, distribution, metabolism, and excretion (ADME) processes that dictate a patient’s exposure to the parent drug and its metabolites. Dysregulation of ADME processes in cancer can alter exposure to administered therapeutics, often resulting in reduced efficacy or likelihood of toxicity. Elucidating the factors contributing to aberrant ADME function, including transporter-mediated MDR common to HCC, provides valuable insights for drug development. Although factors like genetic polymorphisms and nuclear receptor activation have well appreciated effects on ADME gene families, an important but often under-appreciated factor is the post-transcriptional gene regulation (PTGR) of ADME-related proteins by functional microRNAs (miRNAs) derived from the genome.^5^^,^^6^ MiRNAs are double-stranded noncoding RNAs that function as key mediators of gene expression, controlling the protein outcomes during translation through interaction with specific miRNA response elements (MREs) within the 3′-untranslated region (3′UTR) of target mRNA transcripts.^7^ In particular, one strand of the miRNA duplex termed the guide strand (21–23 nt) is incorporated into an RNA-induced silencing complex to identify target MRE sequences through complementary base pairings, primarily by the miRNA seed sequence (nucleotides 2–8). Sequence-specific miRNA-MRE complementarity leads to the repression of translation or promotion of degradation of target transcripts.

PTGR by genome-derived miRNAs has been recognized as a critical mechanism behind large interindividual variations in ADME and pharmacotherapy,^5^^,^^6^^,^^8^ with the let-7 family serving as a good example. The human let-7-5p family miRNAs consist of several isoforms (from let-7a to let-7g plus let-7i) that vary slightly in their sequences (Supplemental Table 1). The let-7 family miRNAs have established roles in regulating genes involved in oncogenic processes, including c-MYC, RAS, CDK6, and LIN28B.^9^, ^10^, ^11^ Meanwhile, several drug-metabolizing enzymes and efflux ABC transporters have been identified as PTGR targets of specific let-7-5p isoforms in human cells, including CYP2J2 by let-7b,^12^ CYP19A1 by let-7f,^13^ ABCC2/MRP2 by let-7c,^14^ ABCC5/MRP5 by let-7a, and ABCC10/MRP7 by let-7a and let-7e.^2^ The broad impact and tumor suppressive function make the let-7 family miRNAs promising candidates to rebalance oncogenic phenotypes and overcome MDR toward developing rational and improved HCC therapies.

Research efforts have highlighted miRNA dysregulation as an important factor in the pathogenesis and MDR of cancer, with the let-7-5p miRNA family being one of the most highly downregulated in HCC.^2^^,^^15^ This downregulation dampens their natural regulatory functions, promoting aberrant protein expression of let-7-5p targets integral to MDR, such as ABCC5/MRP5. Indeed, ABCC5/MRP5 gene expression is inversely correlated with let-7a-5p expression in HCC and is linked to poor prognosis.^15^, ^16^, ^17^, ^18^, ^19^ Therefore, disruption of let-7–controlled PTGR is a major contributor to MDR in HCC, highlighting the potential of miRNA replacement therapy, which seeks to reintroduce or restore let-7-5p species or function to overcome MDR.

To investigate miRNA-based PTGR mechanisms and therapies, the Yu laboratory has developed an innovative RNA bioengineering platform to produce recombinant miRNA molecules.^20^, ^21^, ^22^, ^23^, ^24^ Unlike RNA mimics chemically synthesized in vitro, these bioengineered RNA (BioRNA) agents are constructed in living cells and therefore more accurately capture the structures and modifications of endogenous miRNA.^23^^,^^25^ Most relevantly, a novel bioengineered let-7c-5p agent was effective in inhibiting HCC viability and downregulating ABCC5/MRP5 protein levels in Huh7 cells, increasing intracellular accumulation and anticancer effect of 5-fluorouracil (5-FU).^26^^,^^27^ Recent efforts have optimized this design to create a new generation of fully humanized let-7-5p (BioRNA/let-7-5p) agents.^22^

Although previous research has studied the PTGR functions and anticancer effects of some specific let-7-5p isoforms aforementioned, their comparative effectiveness remains largely unknown. Therefore, the aim of this study was to employ unparalleled BioRNA/let-7-5p agents to delineate the functional differences of 7 major let-7-5p isoforms in the suppression of protein levels of known let-7-5p targets ABCC5/MRP5 and LIN28B and inhibit human HCC cell viability in vitro. Our findings on the variations of different let-7-5p isoforms in controlling target gene expression and HCC cell viability, depending on let-7-5p abundance and its complementarity with specific targets, should provide valuable insights into their comparative effectiveness in PTGR and potential use to combat MDR in HCC.

## 2 Materials and methods

### 2.1 Chemicals and materials

FBS (Cat# 26140079), Dulbecco’s modified Eagle’s medium (DMEM; Cat# 11965092), DMEM/F-12 medium (Cat# 1132033), RIPA lysis buffer (Cat# 89901), Pierce bicinchoninic acid (BCA) protein assay kit (Cat# 23227), opti-MEM reduced serum medium (Cat# 31985062), PBS (Cat# 45000-446), 0.05% trypsin EDTA 1X (Cat# 45000-660) TRIzol reagent (Cat# 15596018), recombinant human epidermal growth factor (Cat# PHG0311), bronchial epithelial cell growth medium Bulletkit with SingleQuot growth supplements (Cat# 11675440), and Lipofectamine 3000 (Cat# L3000001) were purchased from Thermo Fisher Scientific. Protease inhibitor cocktail (Cat# P8340), human fibronectin (Cat# F2006), bovine serum albumin (Cat# A9576), *O-*phosphorylethanolamine (Cat# P0503), and bovine collagen type I (Cat# CLS354231) were purchased from Sigma-Aldrich. TGX Stain-Free FastCast Acrylamide Kit (10%; Cat# 1610183), Clarity Western enhanced chemiluminescence substrates (Cat# 1705061), blotting-grade blocker (Cat# 1706404), polyvinylidene difluoride membranes (Cat# 1620177), Precision Plus All Blue protein standards (Cat# 1610373), and iTaq Universal SYBR Green Supermix (Cat# 1725121) were purchased from Bio-Rad. Eagle’s modified essential medium (Cat# 89428-906), Falcon nontreated tissue culture flasks (Cat# BD353133), Corning treated culture dishes (Cat# 25382-428), NxGen M-MuLV reverse transcriptase (Cat# 97065-184), and premixed dNTP solutions (Cat# 76081-616) were purchased from VWR International. Direct-zol RNA miniPrep kit (Cat# R2061) was purchased from Zymo Research. CellTiter-Glo 2.0 Cell Viability Assay kit (Cat# G9241) was purchased from Promega. Several isoform-specific stem-loop RT and qPCR primers were designed based on the mature hsa-let-7-5p sequences (Supplemental Table 1) and purchased from Integrated DNA Technologies. All other materials, chemicals, and solvents were purchased from VWR, Sigma-Aldrich, or Thermo Fisher Scientific.

### 2.2 Production of BioRNA/let-7-5p molecules

Recombinant or bioengineered miRNA let-7-5p isoforms and control RNA were produced using a human glycyl tRNA-fused hsa-pre-miR-34a carrier, as described most recently.^22^ In brief, target BioRNA were cloned into the target vector, overexpressed in *Escherichia coli*, and purified by anion-exchange fast protein liquid chromatography. BioRNA/let-7-5p agents used in this study were highly pure, as determined by a high-performance liquid chromatography method (≤98%), and showed low endotoxin levels (≤5 EU/*μ*g RNA).

### 2.3 Cell culture

Human Hep3B (HB-8064) and HepG2 (HB-8065), murine Hepa1-6 (CRL-1830) and AML12 (CRL-2254), and healthy human liver epithelial THLE-2 (CRL-2706) and THLE-3 (CRL-3583) cell lines were purchased from American Type Culture Collection (Manassas, VA). Human Huh7 cell line was graciously provided by Dr Baitang Ning.^28^ HepG2 and Hep3B cells were maintained in Eagle’s modified essential medium, Huh7 and Hepa1-6 cells were maintained in DMEM, and AML12 cells were maintained in DMEM/F-12 medium. For THLE-2 and THLE-3 cultures, untreated Falcon flasks were coated overnight with bronchial epithelial cell basal medium supplemented with 0.01 mg/mL fibronectin, 0.03 mg/mL bovine collagen type I, and 0.01 mg/mL bovine serum albumin. THLE-2 and THLE-3 cells were then cultured in bronchial epithelial cell growth medium, which was generated by supplementing bronchial epithelial cell basal medium with SingleQuot reagents according to the manufacturer’s instructions (Lonza Group AG), as well as 5 ng/mL epidermal growth factor and 70 ng/mL *O*-phosphorylethanolamine. All culture media were supplemented with 10% FBS, and all cell lines, verified to be free of mycoplasma contamination, were cultured in a humidified incubator (37 °C, 5% CO_2_). Cell counting for all experiments was performed using the Countess II FL system (Thermo Fisher Scientific).

### 2.4 Protein isolation and Western blot analysis

All cells used to compare the basal levels of proteins of interest were cultured in specified vessels, not treated with any agent, and harvested at 80% confluence. Human Huh7, HepG2, Hep3B, and THLE-2 cells used in experiments assessing the modulation of ABC transporter and LIN28B protein levels were seeded into 6-well plates (500,000 cells/well) and transfected with 15 nM of biologic let-7-5p or control RNA, or Lipofectamine vehicle only for 72 hours. Cells were then lysed in RIPA buffer containing protease inhibitor cocktail and remained on ice for 30 minutes before thorough mixing and centrifugation. Supernatant was collected for each sample, and protein concentration was quantified using the Pierce BCA protein assay kit. Protein samples (30 *μ*g) were loaded into individual lanes of a 10% TGX stain-free SDS-PAGE gel, electrophoretically separated, and imaged with the Chemidoc MP system (Bio-Rad) for total protein determination before being transferred to polyvinylidene difluoride membrane using the Trans-Blot Turbo Transfer System (Bio-Rad). Membranes were treated with 5% blotting-grade blocker for 2 hours (25 °C) to minimize background signal and then incubated overnight (4 °C) with primary antibodies against c-MYC (1:1000; Cell Signaling Technology Cat# 5605), PD-L1 (1:1000; Cell Signaling Technology Cat# 13684S), CDK6 (1:200; Santa Cruz Biotechnology; Cat# sc-177), LIN28B (1:1000; Cell Signaling Technology; Cat# 11965), ABCC1/MRP1 (1:2500; Proteintech; Cat# 67228-1-lg), ABCC2/MRP2 (1:500; Santa Cruz Biotechnology; Cat# sc-518048), ABCC3/MRP3 (1:1000; Cell Signaling Technology; Cat# 39909), ABCC4/MRP4 (1:1000; Abcam; Cat# ab15602), ABCC5/MRP5 (1:200; Santa Cruz Biotechnology; Cat# sc-376965), or *β*-actin (1:1000; Sigma-Aldrich; Cat# A5441). Membranes were then incubated in one of the following HRP-linked secondary antibodies for 2 hours (25 °C): anti-mouse IgG (1:3000; Cell Signaling Technology; Cat# 7076), anti-rabbit IgG (1:10,000; Jackson ImmunoResearch; Cat# 111-035-003), anti-rat IgG (1:3000; Cell Signaling Technology; Cat# 7077). Membranes were then briefly incubated in a 1:1 mixture of Clarity Western enhanced chemiluminescence substrates, and protein images were obtained with the Chemidoc MP system. Image Lab software (Bio-Rad) was used to quantify target protein band intensity, which was normalized to total protein and *β*-actin levels within the corresponding sample, with control RNA set as 1.0. All BioRNA/let-7-5p treatments were performed in biological triplicate (N = 3), with individual protein lysate samples being pooled for Western blot analyses that were conducted technically at least twice.

### 2.5 RNA extraction and stem-loop reverse transcription, quantitative real-time PCR analyses

Human Huh7, HepG2, Hep3B, and THLE-2 cells were seeded into 12-well plates (250,000 cells/well) and transfected with 15 nM of BioRNA/let-7-5p or control RNA, or Lipofectamine vehicle for 48 hours in biological triplicate (N = 3/group). Direct-zol RNA miniPrep kit (Zymo Research) was used to isolate total RNA from treated cells, and RNA concentration was determined using a Tecan Spark microplate reader. Specific stem-loop RT and qPCR primers were designed for individual let-7-5p isoforms as detailed in Supplemental Table 1. Total RNA (300 ng) was reverse-transcribed to cDNA using NxGen M-MuLV reverse transcriptase and corresponding stem-loop RT primers. Quantitative PCR analyses utilized sequence-specific forward primers for each let-7-5p isoform and its corresponding vehicle and RNA controls. U6 small RNA levels were separately assessed for each sample using U6-specific primers (Supplemental Table 1). Real-time analyses were prepared using the iTaq Universal SYBR Green Supermix according to the manufacturer’s instructions and performed on a CFX96 Touch Real-Time PCR System (Bio-Rad). For each sample, levels of let-7-5p isoforms were normalized to their respective U6 small RNA levels, and the formula 2^–ΔΔCT^ was used to calculate the fold change of individual let-7-5p isoforms. Each biological sample was analyzed with 2 technical replicates.

### 2.6 Cell growth and viability assays

Human Huh7, Hep3B, and HepG2 cells were seeded into 96-well plates at a density of 7500 cells/well and transfected with BioRNA/let-7-5p or control RNA (15 nM), or Lipofectamine vehicle for 72 hours (N = 3/group). An Incucyte Live-Cell Imaging and Analysis system (Sartorius AG) was used to determine cell confluency values for every sample at 4-hour intervals, allowing construction of cell growth curves. Absolute cell confluency values were fit to the Malthusian exponential growth equation, Y=Y0∗exp/(k∗x), using GraphPad Prism. Kinetic parameters were estimated for each treatment, including the starting confluency (Y0), rate constant (k), and doubling time (ln(2)/k). Cells were removed from the incubator after 72 hours, and the CellTiter-Glo 2.0 Cell Viability Assay Kit was used to further validate cell viability.

### 2.7 Statistical analysis

All values are expressed as mean ± SD. Statistical analysis was performed using one- or two-way ANOVA with Bonferroni or Tukey post hoc tests, where appropriate (GraphPad Prism). Differences between groups were considered significant when the probability value was less than .05 (*P* < .05).

## 3 Results

### 3.1 Identification of proper liver cell lines for the study of let-7-5p–controlled target gene expression

**Fig. 1:**
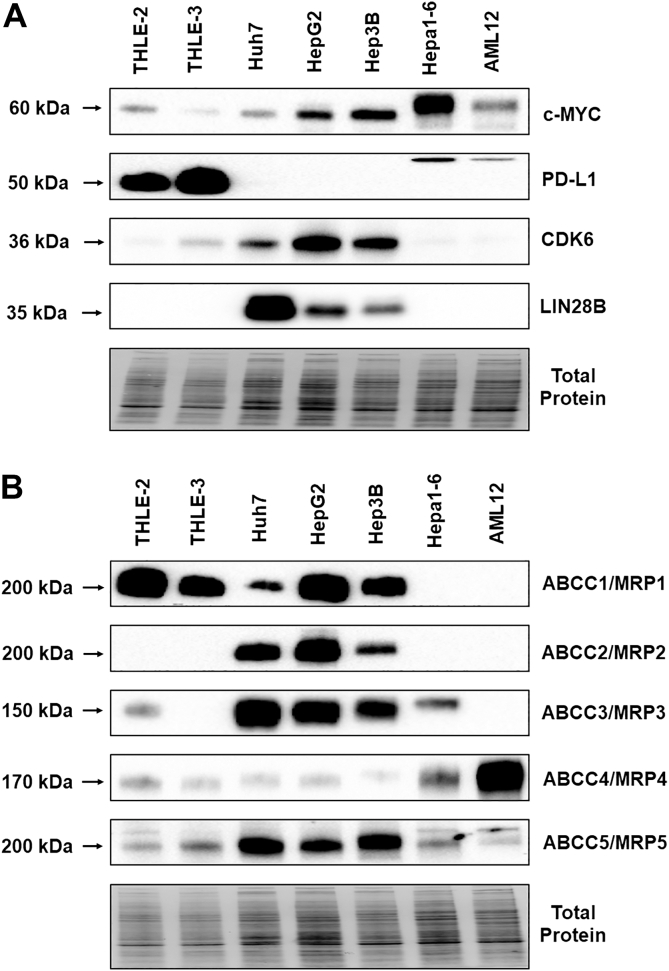
Comparison of basal levels of several proteins in a panel of liver cell lines, including human (Huh7, HepG2, and Hep3B) and murine (Hepa1-6 and AML12) hepatocellular carcinoma cells, as well as 2 derived from healthy human liver epithelium (THLE-2 and THLE-3), without any treatments. (A) c-MYC, PD-L1, CDK6, and LIN28B are known let-7-5p targets important in cancer. (B) Common multidrug resistance transporters ABCC1-5 (MRP1-5), in which ABCC5 is a direct target of let-7-5p. Western blot analyses were conducted with selective antibodies.

**Fig. 2:**
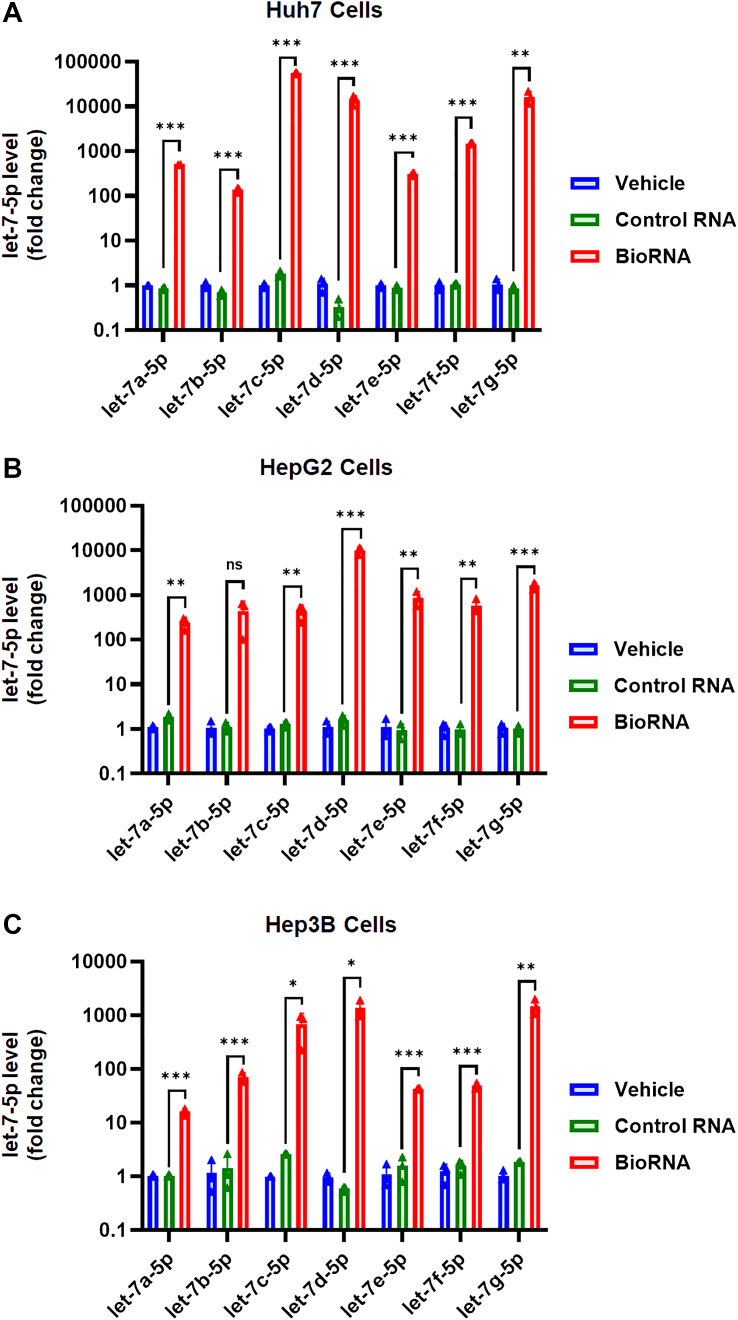
BioRNA/let-7-5p agents are selectively processed to their respective let-7-5p isoforms in (A) Huh7, (B) HepG2, and (C) Hep3B cells. Selective stem-loop RT real-time qPCR methods were used to measure individual let-7-5p isoforms, and their levels were normalized to U6 levels in corresponding samples. ∗∗∗*P* < .001; ∗∗*P* < .01; ∗*P* < .05; and ns, not significant (one-way ANOVA with Bonferroni post hoc tests). All values are mean ± SD (N = 3 biological replicates per treatment group).

To identify suitable cell models to investigate functional differences of let-7-5p isoforms, the basal protein levels of several known and predicted let-7-5p targets were compared across 7 liver cell lines without any treatments. Overall, liver cells showed variable basal protein levels of cancer-related (c-MYC, CDK6, LIN28B, PD-L1) and ABC transporter (ABCC1-5/MRP1-5) targets (Fig. 1). Human HCC cells (Huh7, HepG2, Hep3B) showed basal expression of several validated let-7-5p targets important in cancer (c-MYC, CDK6, LIN28B) but did not show expression of human PD-L1 protein (Fig. 1A). Notably, basal expression of LIN28B protein was highly variable across human HCC cells, with Huh7 exhibiting basal LIN28B levels that were dramatically higher than HepG2 and Hep3B. Densitometric analysis of LIN28B revealed relative intensity values across human HCC cells, with HepG2 and Hep3B exhibiting 38% and 27% of the present Huh7 LIN28B signal, respectively, normalized to corresponding total protein (Fig. 1A). Conversely, Huh7 showed the lowest basal protein levels of both c-MYC and CDK6 across human HCC cells. Huh7, HepG2, and Hep3B also demonstrated the highest basal protein expression of several transporter targets (ABCC2/MRP2, ABCC3/MRP3, ABCC5/MRP5). Basal transporter levels were comparable overall, with the lowest ABCC2/MRP2 levels detected in Hep3B cells. Observed basal ABCC1/MRP1 protein levels were comparably high across human HCC cells and transformed healthy human liver epithelial cells (THLE-2 and THLE-3), with the highest basal levels detected in HepG2 and THLE-2 and the lowest observed in Huh7 (Fig. 2B). Uniquely, high PD-L1 protein levels were detected in THLE-2 and THLE-3 cells with minimal or no basal protein expression of other targets (Fig. 1). Both murine liver carcinoma cell lines (Hepa1-6 and AML12) demonstrated high basal protein expression of c-MYC and ABCC4/MRP4, with Hepa1-6 also exhibiting some basal expression of ABCC3/MRP3. Basal expression of ABCC4/MRP4 protein was detected in all cell lines but was the most highly expressed in AML12 cells (Fig. 1). The basal protein expression of the known let-7-5p target ABCC5/MRP5 was detected across all tested cell lines but showed the highest expression in human HCC cells (Huh7, HepG2, Hep3B) (Fig. 1B).

### 3.2 BioRNA/let-7-5p agents are processed to target let-7-5p isoforms in human HCC cells

Human HCC cells (Huh7, Hep3B, HepG2) with high basal protein levels of let-7-5p targets (LIN28B and ABCC5/MRP5) were, thus, selected for further investigation. To assess whether BioRNA/let-7-5p agents were effectively processed to their respective let-7-5p isoforms, stem-loop reverse transcription, quantitative real-time PCR (RT-qPCR) analyses were employed to measure individual let-7-5p miRNA levels following transfection with BioRNA/let-7-5p for 48 hours. Levels of each let-7-5p miRNA were measured by individual stem-loop RT-qPCR analyses with sequence-specific primers (Supplemental Table 1). The results showed that BioRNA/let-7-5p treatments resulted in higher levels of corresponding let-7-5p isoforms in Huh7, HepG2, and Hep3B cells, with some consistent differences observed across individual cell lines and isoforms (Fig. 2). Across all 3 human HCC cells, 17 out of 21 BioRNA/let-7-5p treatments exhibited 100-fold or greater change of corresponding let-7-5p miRNA, with many treatments also far exceeding this value. All 4 RT-qPCR analyses that fell short of this 100-fold change threshold were observed in Hep3B cells, which consistently showed overall lower fold change values compared to equivalent experiments in Huh7 and HepG2 cells. In Huh7 cells, let-7c-5p, let-7d-5p, and let-7g-5p all demonstrated higher than 10,000-fold change following treatment, with let-7c-5p being the most highly detected (Fig. 2A). In HepG2 cells, let-7d-5p showed the highest fold change near 10,000-fold, let-7g-5p showed the second highest near 1500-fold, and remaining isoforms exhibited increases between 100- and 1000-fold (Fig. 2B). Although overall findings in Hep3B cells showed comparatively lower fold changes, the comparative trend between individual isoforms was consistent with Huh7 cells, with the highest increase observed for let-7c-5p, let-7d-5p, and let-7g-5p (over 500–1000 fold) treatments (Fig. 2, A and C). These findings demonstrate that let-7-5p isoforms are successfully released from BioRNA/let-7-5p agents and accumulated to varying degrees in human HCC cells, with let-7d-5p consistently exhibiting one of the highest fold increases across all cell lines.

### 3.3 Comparison of the effectiveness of individual let-7-5p isoforms to downregulate protein levels of ABCC5 and LIN28B in human HCC cells

**Fig. 3:**
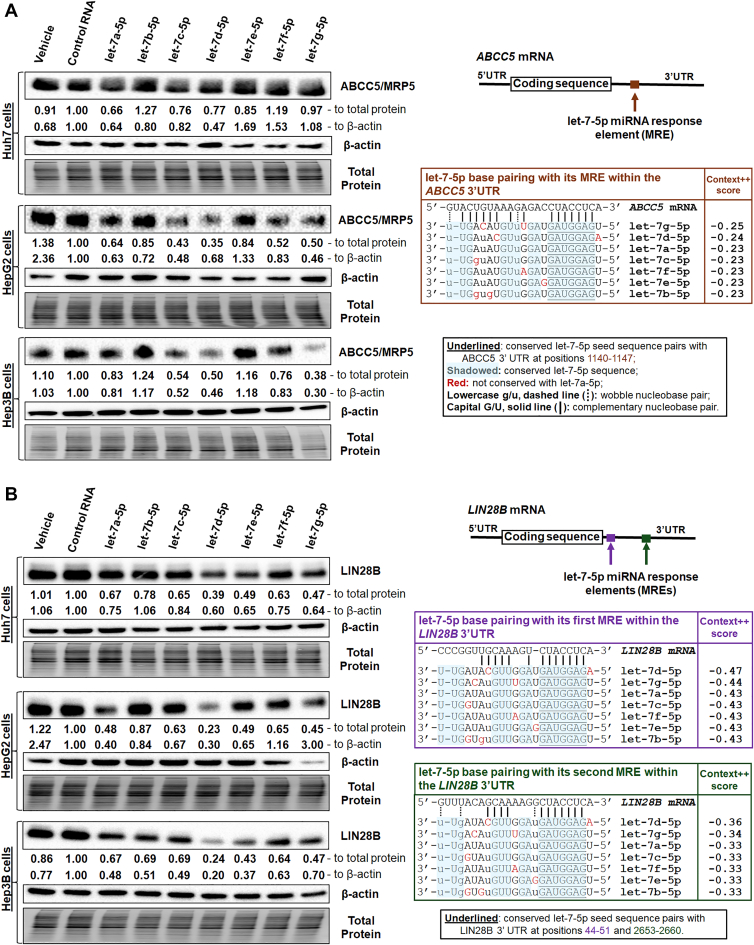
Comparison of the effectiveness of various let-7-5p isoforms to downregulate protein levels of (A) ABCC5/MRP5 and (B) LIN28B in multiple human liver cells. Huh7, HepG2, and Hep3B cells were treated with 15 nM of bioengineered let-7-5p or control RNA or vehicle for 72 hours, and Western blot analyses were performed to determine protein levels. Representative blots are shown. Protein band densities were normalized to corresponding total protein or *β*-actin levels, with the control RNA group set as 1.0. Interactions of individual let-7-5p isoforms with respective MREs within (A) ABCC5/MRP5 and (B) LIN28B 3′UTR have been illustrated. Individual let-7-5p isoforms are listed according to the Context scores (TargetScan).

To determine the effectiveness of individual BioRNA/let-7-5p isoforms in modulating protein levels of known let-7-5p targets ABCC5/MRP5 and LIN28B, immunoblot analyses were performed in Huh7, HepG2, and Hep3B cells transfected with BioRNA/let-7-5p or control RNA. Across all cells, multiple BioRNA/let-7-5p treatments successfully downregulated ABCC5/MRP5 protein levels when compared to control RNA (Fig. 3A). In Huh7 cells, let-7a-5p, let-7c-5p, and let-7d-5p treatments showed the largest decreases in ABCC5/MRP5 protein levels (34%, 24%, and 23% respectively), as compared to control RNA (Fig. 3A). In HepG2 cells, let-7c-5p, let-7d-5p, and let-7g-5p treatments showed the strongest downregulation of ABCC5/MRP5 protein levels (57%, 65%, and 50% respectively7) whereas let-7a-5p exhibited a modest suppression of ABCC5/MRP5 protein comparable to Huh7 (36%) (Fig. 3A). Similar results were observed in Hep3B cells, where let-7c-5p, let-7d-5p, and let-7g-5p treatments strongly reduced ABCC5/MRP5 protein levels (46%, 50%, and 62% respectively) compared to control RNA, with let-7a-5p treatment showing only minor suppression of ABCC5/MRP5 protein levels (17%), distinct results in Huh7 and HepG2. Similarly, BioRNA/let-7g-5p treatment was among the most effective to downregulate ABCC5/MRP5 in HepG2 and Hep3B cells but notably showed minimal effects in Huh7 cells (Fig. 3A). On the other hand, there was no or minimal reduction of ABCC2/MRP2 or ABCC4/MRP4 protein levels in HepG2, Hep3B, or Huh7 cells by any of the 7 major let-7-5p isoforms examined in this study (Supplemental Fig. 1).

Multiple BioRNA/let-7-5p isoforms also proved effective to downregulate LIN28B protein levels in human HCC cells (Fig. 3B). Out of all tested isoforms, BioRNA/let-7d-5p consistently showed the strongest effects to reduce LIN28B protein levels, by about 61%, 77%, and 76% in Huh7, HepG2, and Hep3B cells, respectively, as compared to control RNA (Fig. 3B). Although BioRNA/let-7d-5p was consistently the most effective, all treatments successfully downregulated LIN28B to variable degrees. Consistent with observed results for ABCC5/MRP5, BioRNA/let-7b-5p was the least effective treatment to reduce LIN28B protein levels but still showed decreases of 22%, 13%, and 31% in Huh7, HepG2, and Hep3B cells, respectively (Fig. 3B). All other isoforms reduced LIN28B protein levels by about 33%, 35%, and 31% in Huh7, HepG2, and Hep3B cells, respectively. Collectively, these results demonstrate that several biologic let-7-5p isoforms effectively downregulate protein levels of ABCC5/MRP5 and LIN28B across different human HCC cells, with some variability. Additionally, our findings illustrate consistent differences in the efficacy of individual let-7-5p isoforms to modulate protein levels of these targets, with let-7c-5p and let-7d-5p proving the most consistently effective to suppress ABCC5/MRP5 in 3 distinct human HCC cells. Similarly, BioRNA/let-7d-5p was the most effective treatment to downregulate LIN28B protein, with a high degree of consistency across various cell lines (Fig. 3).

Additionally, interactions were illustrated between individual let-7-5p isoforms and MREs within the 3′UTR of ABCC5/MRP5 and LIN28B mRNA transcripts (Fig. 3). Each let-7-5p isoform shares a conserved seed sequence on its 5′ end, mediating complementary 8mer interactions at each MRE within ABCC5/MRP5 and LIN28B mRNA transcripts (Fig. 3). Although all let-7-5p isoforms share complementary 8mer sites at the illustrated MREs, each isoform may differ in their number of additional complementary nucleobase pairings and G-U wobble base pairings upstream of the conserved seed sequence.^29^ Notable differences are also present adjacent to the conserved seed sequence, with let-7d-5p possessing a unique adenosine as the first nucleobase on its 5′ end (Fig. 3). Individual let-7-5p isoforms were ordered at each MRE according to their TargetScan Context++ scores, with the lowest scores representing the most favorable miRNA/mRNA interaction. Context++ scores are calculated as the sum of 14 distinct features that characterize the MRE and its surrounding context, accounting for factors such as seed match identity (eg, 8mer, 7mer-m8), supplementary nonseed base pairing, local AU abundance at the mRNA target, nucleotide identity at positions 1 and 8 of the miRNA, and thermodynamic stability of seed-pairing.^29^ Overall, greater degrees of increase in let-7-5p isoforms (Fig. 2) and their complementarities with target transcripts (Fig. 3) forecast the strength of reduction of targeted protein levels (eg, LIN28B by let-7d-5p) (Fig. 3).

### 3.4 BioRNA/let-7-5p agents are processed to respective let-7-5p isoforms and variably regulate ABCC5/MRP5 protein levels in THLE-2 cells

**Fig. 4:**
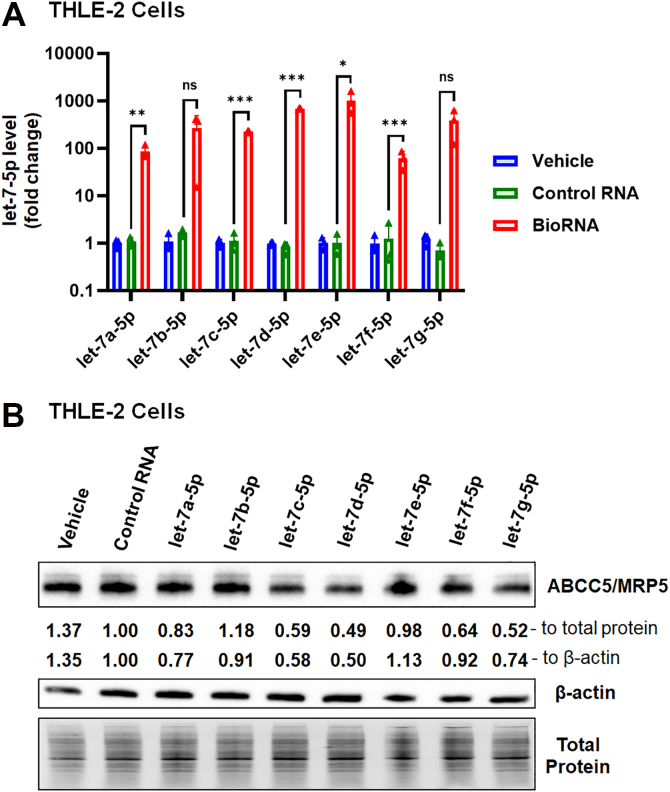
Release of let-7-5p isoforms from BioRNA/let-7-5p molecules and their effectiveness to suppress ABCC5/MRP5 protein levels in THLE-2 cells. (A) Levels of let-7-5p isoforms were measured by selective stem-loop RT real-time qPCR methods and normalized to respective U6 levels. ∗∗∗*P* < .0.001; ∗∗*P* < .0.01; ∗*P* < .0.05; and ns, not significant (one-way ANOVA with Bonferroni post hoc tests). Values are mean ± SD (N = 3 biological replicates per treatment group). (B) Western blot analyses were performed to determine ABCC5/MRP5 protein levels in THLE-2 cells after 72 hours of transfection with 15 nM of RNA or vehicle. Representative blots are shown. Protein band densities were normalized to corresponding total protein levels or *β*-actin, with the control RNA group set as 1.0. 5′UTR, 5′-untranslated region.

Human liver epithelial cells (THLE-2) were further utilized to assess the efficiency of BioRNA/let-7-5p processing and regulation of ABCC5/MRP5 protein levels. Stem-loop RT-qPCR analyses demonstrated selective processing of BioRNA/let-7-5p agents to their corresponding let-7-5p isoforms, with 5 of 7 isoforms exhibiting greater than 200-fold change (Fig. 4A). Interestingly, observed fold change values for several isoforms in THLE-2 (most notably let-7a-5p, let-7b-5p, let-7e-5p) were of greater magnitude than those observed in Hep3B cells, but generally less than those observed in Huh7 and HepG2 cells. However, stem-loop RT-qPCR analyses in THLE-2 cells showed a comparative trend in the fold change of individual let-7-5p isoforms that was distinct from observations in human HCC cells. Unlike Huh7, HepG2, and Hep3B cells, which consistently exhibited large increases in detected let-7c-5p, let-7d-5p, and let-7g-5p levels, THLE-2 cells demonstrated comparatively modest increases of these isoforms (Fig. 4A). Additionally, THLE-2 cells showed an increase of let-7e-5p species greater than 500-fold, exceeding the fold change values observed for this isoform in Huh7 and Hep3B cells.

The comparative effectiveness of let-7-5p isoforms to regulate ABCC5/MRP5 protein levels in THLE-2 was investigated following transfection with individual BioRNA/let-7-5p agents for 72 hours. Consistent with the results obtained from multiple human HCC cells, let-7-5p isoforms showed variable degrees of efficacy to suppress ABCC5/MRP5 protein levels in THLE-2, with let-7c-5p (41%), let-7d-5p (51%), and let-7g-5p (48%) showing greater extents of downregulation as compared to control RNA (Fig. 4B).

### 3.5 Bioengineered let-7-5p isoforms inhibit the growth of human HCC cells to variable degrees

**Fig. 5:**
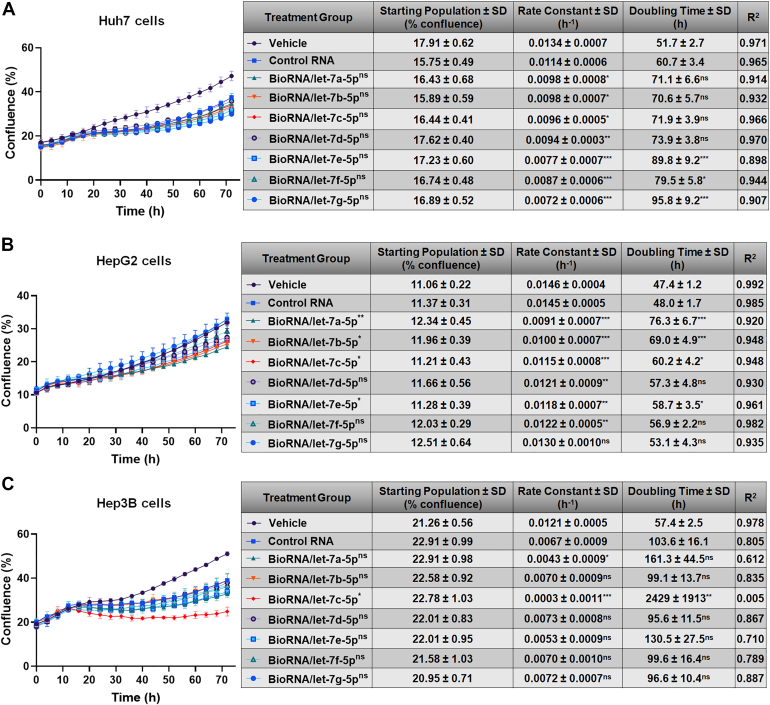
Efficacy of individual let-7-5p isoforms to inhibit (A) Huh7, (B) HepG2, and (C) Hep3B cell viability over time. Cell confluency was monitored for 72 hours with an Incucyte Live-Cell Imaging and Analysis System, after treatment with 15 nM of biologic let-7-5p or control RNA or vehicle. Cell confluency values were measured at 4-hour intervals and used to construct cell confluency growth curves for each treatment. ∗*P* < .05; and ns, not significant; let-7c-5p compared to control RNA (two-way ANOVA with Tukey post hoc tests). Data were fit to the exponential (Malthusian) growth equation, Y=Y0∗exp/(k∗x), and kinetic parameters were estimated, including the starting confluency (Y0), rate constant (k), and doubling time (ln(2)/k). ∗∗∗*P* < .001; ∗∗*P* < .01; ∗*P* < .05; and ns, not significant; individual let-7-5p isoforms compared to control RNA (one-way ANOVA with Bonferroni post hoc tests). All values are mean ± SD (N = 3 biological replicates per treatment group).

Cell growth and viability experiments were carried out to investigate the efficacy of biologic let-7-5p isoforms to inhibit the viability of human HCC cells (Huh7, HepG2, Hep3B). Following transfection with BioRNA/let-7-5p agents, cell confluency values were measured over 72 hours and used to assess cell growth over time for each treatment. Data fitted well to the Malthusian exponential growth equation, Y=Y0∗exp/(k∗x), and relevant kinetic parameters were estimated (Fig. 5). The results showed that multiple biologic let-7-5p agents effectively inhibited viability of human HCC cells, with key differences in their comparative effectiveness observed across different HCC cells (Fig. 5). In Huh7 and HepG2 cells, all BioRNA/let-7-5p treatments exhibited increased doubling time (ln(2)/k) and decreased rate constant (k) values compared to control RNA (Fig. 5, A and B). For Huh7 cells, let-7e-5p, let-7f-5p, and let-7g-5p treatments resulted in the largest changes in these parameters, while let-7a-5p, let-7b-5p, and let-7c-5p treatments showed the largest changes in HepG2 cells (Fig. 5, A and B). Results in Hep3B cells showed interesting differences, where only let-7a-5p, let-7c-5p, and let-7e-5p treatments exhibited increased doubling time compared to control RNA; among them, let-7c-5p was the most effective isoform (Fig. 5C). It is noted that Hep3B and Huh7 cells treated with control RNA also exhibited large differences in doubling time when compared to vehicle control, whereas no difference was observed between control RNA and vehicle treatments in HepG2 cells (Fig. 5C).

**Fig. 6:**
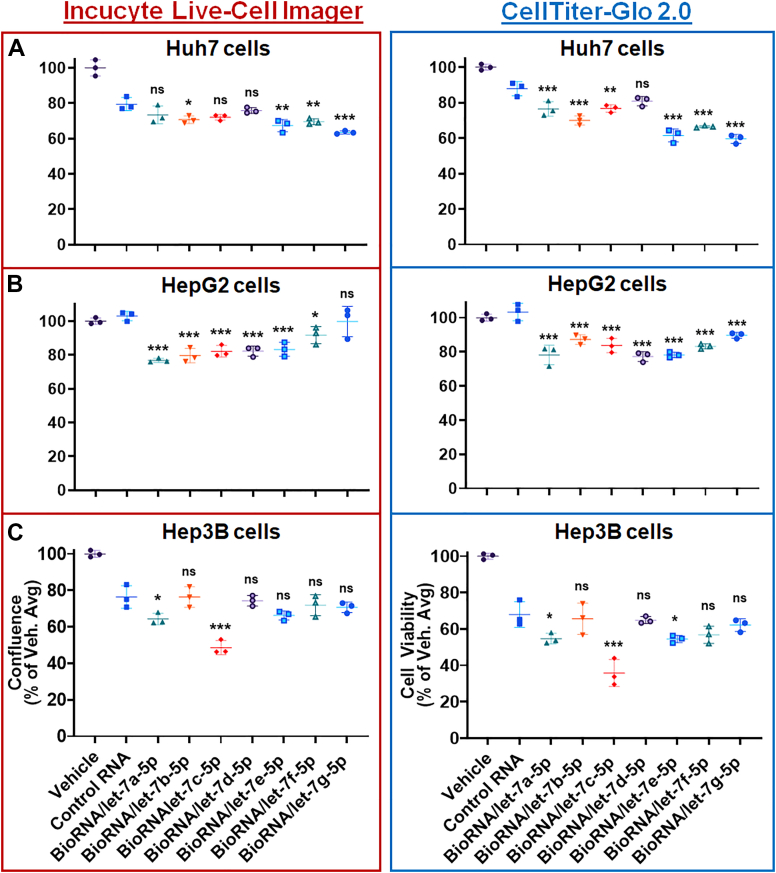
Comparison of Incucyte Live-Cell Imaging method (left) and CellTiter Assay (right) to determine BioRNA/let-7-5p efficacy to inhibit (A) Huh7, (B) HepG2, and (C) Hep3B cell viability, after 72-hour treatment with 15 nM of RNA. Vehicle treatments were included as additional controls. ∗∗∗*P* < .001; ∗∗*P* < .01; ∗*P* < .05; and ns, not significant; individual BioRNA/let-7-5p isoforms compared to control RNA (one-way ANOVA with Bonferroni post hoc tests). All values are mean ± SD and represented as the percentage (%) of vehicle control (N = 3 biological replicates per treatment group).

At the 72-hour endpoint, cell viability values were also measured with CellTiter-Glo 2.0 Assay (Fig. 6). Across all 3 cell lines and individual treatments, 72-hour endpoint confluence values were generally consistent with results obtained from CellTiter-Glo cell viability assay and Incucyte Live-Cell Imaging and Analysis method (Fig. 6). Cell viability analyses showed differences between treatment groups that mirror changes to doubling time (ln(2)/k) and rate constant (k) as described above. Consistent with changes to estimated parameters, BioRNA/let-7e-5p, let-7f-5p, and let-7g-5p treatments exhibited the strongest effects against Huh7 cell viability (Figs. 5A and 6A). Some small differences were observed in HepG2 cells, with BioRNA/let-7d-5p and let-7e-5p treatments exhibiting stronger inhibition of endpoint viability compared to BioRNA/let-7b-5p, which showed a larger change in estimated parameters (Figs. 5B and 6B). Endpoint cell viability results in Hep3B cells also reflect the observed changes in estimated kinetic parameters, with BioRNA/let-7c-5p demonstrating the strongest inhibition of Hep3B cell viability compared to control RNA (Figs. 5C and 6C). Overall, growth curve and endpoint cell viability analyses showed that multiple let-7-5p isoforms are effective in inhibiting the viability and growth of different human HCC cells, with some key differences between isoforms and across cell lines.

## 4 Discussion

Multiple efflux transporters confer resistance to standard HCC therapies, with several studies supporting the role of efflux transporters such as ABCC2/MRP2, ABCC4/MRP4, and ABCC5/MRP5 in mediating resistance to drugs like sorafenib, 5-FU, and oxaliplatin.^18^^,^^27^^,^^30^ Therapeutic resistance may occur through direct efflux of administered drug as well as indirect factors related to endobiotic transport. Although ABCC5/MRP5 does not readily transport sorafenib, it is strongly induced in sorafenib-resistant HCC cells and provides protection against sorafenib-induced ferroptosis by promoting the expression of solute carrier SLC7A11.^18^ In HCC and many other types of cancers, the let-7 family is among the most consistently downregulated species of miRNAs, with repressed let-7 miRNA levels associated with increased expression of genes implicated in MDR (ABCC1-5/MRP1-5).^2^^,^^15^^,^^31^, ^32^, ^33^ Although several studies have demonstrated the potential of let-7-5p to regulate ABC transporters (ABCC2, ABCC4, ABCC5) and resensitize multiple cancers to therapy, the present study found minimal effects of let-7-5p isoforms on the protein outcomes of ABCC2/MRP2 or ABCC4/MRP4 in HCC cells in vitro, while exhibiting functional differences in regulating ABCC5 protein levels.

HCC progression and persistent MDR phenotype resulting from low let-7-5p expression is due in part to RNA-binding protein LIN28B, which is overexpressed in HCC and actively represses maturation of endogenous let-7 miRNA species through specific interaction with their primary (pri-) and precursor (pre-) miRNAs, preventing proper processing by the Drosha microprocessor and Dicer complexes, respectively.^34^, ^35^, ^36^ LIN28B, an established target of let-7-5p,^37^, ^38^, ^39^ also promotes 3′ polyuridination of pre–let-7 through terminal uridylyl transferases, suppressing let-7-5p levels and therefore promoting overexpression of ABCC5/MRP5. MiRNA let-7-5p can also regulate the expression of LIN28B through PTGR, a well established feedback loop also supported by this study that interrogated the comparative efficacy of individual let-7-5p isoforms to repress protein levels of validated targets LIN28B and ABCC5 in HCC cells.

BioRNA/let-7d-5p treatment showed the strongest and most consistent downregulation of LIN28B protein levels across 3 human HCC cell lines. The superior efficacy of let-7d-5p to regulate LIN28B is directly correlated with its highest degree of target complementarity outside of the seed sequence for both MREs within the LIN28B 3′UTR. Illustrated alignments confirm that let-7d-5p is the only isoform to form a stable C-G nonseed pair in both LIN28B MREs, distinct from the U-G wobble base pair present at this position for all other let-7-5p isoforms. This interaction, specific to let-7d-5p, fosters a higher degree of target complementarity outside the seed region^40^ and provides a clear explanation for its superior regulatory strength observed in this study. Let-7d-5p also possesses a unique adenosine at its 5′ end, which does not prevent 8mer interaction with MRE’s in LIN28B or ABCC5/MRP5 transcripts, but does influence its natural biogenesis^41^ and might also impact how well our BioRNA/let-7-5p agents are processed to target let-7-5p isoforms. Despite BioRNA/let-7d-5p being the most effective isoform to reduce LIN28B protein levels, it was not the most effective to inhibit HCC cell viability. This indicates that although the degree of LIN28B repression is important for HCC viability, let-7 miRNA exerts anticancer effects through a broader network of targets that may differ between individual cell lines, emphasizing the importance of paired phenotypic assessments as performed in this study.

Central to the present study was the evaluation of BioRNA/let-7-5p agents to repress protein levels of protective efflux transporter ABCC5/MRP5. In particular, let-7c, let-7d, and let-7g-5p were identified as more effective suppressors of ABCC5/MRP5 protein levels across distinct HCC cell backgrounds. These results further demonstrate the potential of BioRNA/let-7-5p for use in rational combination therapy and provide a strong mechanistic rationale for coadministration of let-7-5p with existing drugs subject to efflux by ABCC5/MRP5, such as 5-FU.^27^ Considering the effective downregulation of LIN28B in Huh7 by BioRNA/let-7g-5p, the observed lack of regulation of ABCC5/MRP5 by this isoform is likely to be target-specific but not necessarily correlated with let-7-5p MRE complementarity. Let-7g-5p can form the largest number of complementary nucleobase pairings outside of the seed sequence with the MRE within ABCC5. Most notably, let-7g-5p contains a cytosine near its 3′ end, where all other isoforms have uracil, allowing formation of a complementary C-G pairing where all other isoforms have a U-G wobble base pair, supporting its high degree of regulatory strength against ABCC5 observed in HepG2 and Hep3B cells. These results demonstrate a separation between the factors driving anticancer effects of BioRNA/let-7-5p agents from those governing regulation of MDR-related targets such as ABCC5/MRP5, dependent on the cellular microenvironment.

Overall, Huh7 cells exhibited a lower magnitude of ABCC5/MRP5 suppression than HepG2 and Hep3B cells despite showing the highest fold change of let-7-5p miRNAs. Potential factors explaining this lower PTGR magnitude specific to ABCC5/MRP5 in Huh7 are the variable basal expression of individual let-7-5p isoforms and high basal expression of IGF2BP1, an RNA-binding protein with established influence on MDR that may stabilize mRNA transcripts of efflux transporters like ABCC5/MRP5 or limit accessibility to let-7 MREs within the 3′UTR.^42^^,^^43^ The role of IGF2BP1 in protecting ABCC5/MRP5 mRNA was evident in a recent study utilizing a specific small molecule inhibitor of IGF2BP1, which suppressed ABCC5/MRP5 gene expression.^43^ Besides the variable basal levels of individual let-7-5p isoforms leading to distinct degrees of increase in specific let-7-5p isoforms upon BioRNA treatments, cell lines may also differ in their basal levels of protein targets or endogenous factors that dampen let-7-5p regulatory outcomes. In this study, ABCC5/MRP5 was found to be expressed at consistent levels across human HCC cells, whereas LIN28B expression was noticeably higher in Huh7 cells. Variable basal expression of let-7-5p miRNA, their corresponding protein targets, and related endogenous factors like IGF2BP1 underlie the high heterogeneity observed in HCC, providing context to the cell line–specific differences in let-7-5p treatment outcomes observed in this study. Additionally, the transfection efficiencies differ a lot among these HCC cell lines, which likely contributes to the variability of particular effects.

Much like the unique subpopulations that comprise microenvironments of a heterogeneous tumor, individual HCC cell lines possess distinct cellular morphologies and genetic backgrounds that foster variable energy metabolism and modulate responses to exposome or stress, such as xenobiotics and hypoxia. Further, the exosomes or extracellular vesicles released by HCC cells to facilitate cell-cell communications and microenvironments may differ much in their active components, including exosomal miRNAs,^44^ highlighting a potential underlying factor that may contribute to variable let-7-5p effectiveness across individual HCC cell lines observed in this study. Under hypoxic conditions, hypoxia-inducible factor-1 *α* signaling largely affects the size, quantity, and composition of exosomes^45^ in which the upregulation of exosomal miR-155-5p has been revealed to promote angiogenesis in endothelial cells.^46^ On the other hand, exosomal oncogenic miR-155-5p derived from hypoxic tumor-associated macrophages promotes the progression of renal cell carcinoma.^47^ Rather, it is noteworthy that exosomes represent a novel system for the delivery of therapeutic RNA molecules, including miRNAs.^48^

To robustly assess the effect of BioRNA/let-7-5p treatments on HCC cell viability, 2 methods were used in this study. The Incucyte system offers real-time label-free imaging and analysis of cell confluence over time, allowing us to capture subtle changes in growth kinetics (rate constant and doubling time) not captured by assays at a single time point. Meanwhile, the live-cell imaging technology was complemented with the biochemical CellTiter-Glo 2.0 assay, which is based on measuring cellular ATP levels that reflect changes in cell proliferation and cellular energy state.^49^^,^^50^ By utilizing both imaging and biochemical information to assess HCC cell viability, the disadvantages of each approach are minimized to conduct a more complete and unbiased assessment of treatment effects. Consistent with our recent findings,^51^ orthogonal imaging and biochemical assays agreed well with one another, revealing functional differences among individual let-7-5p isoforms in modulating cell viability. This rigorous dual-pronged approach showing high concordance between orthogonal analyses supports the validity of our findings as genuine pharmacological effects of BioRNA/let-7-5p molecules.

Although the present study interrogates the comparative effects of 7 major human let-7-5p isoforms in the regulation of target gene expression and HCC cell viability by using unique BioRNA/let-7-5p agents, these findings are limited to particular in vitro cell line models. The translational potential warrants further investigations with more clinically relevant in vivo or ex vivo models. Indeed, many association or correlation studies reported previously^2^^,^^52^, ^53^, ^54^, ^55^, ^56^ have demonstrated the value of using human specimens for identifying specific miRNAs and defining their significance in the regulation of particular drug-metabolizing enzymes or ABC transporters, as well as developing new miRNA-based therapeutics. On the other hand, our previous studies have extended some in vitro observations to in vivo studies to reveal the translational potential of miRNA replacement therapy in disease animal models,^21^^,^^26^^,^^51^^,^^57^, ^58^, ^59^, ^60^, ^61^ in which pharmacological synergism between an onco-suppressive miRNA with a coadministered drug may be attributable to interactions occurring at both pharmacodynamic and pharmacokinetic levels.^6^ Moreover, the present study is also limited to the use of a gain-of-function approach, which is appropriate when examining the miRNAs (eg, let-7-5p) downregulated and their targets of interest (eg, ABCC5 and LIN28B) upregulated in the model systems (eg, HCC cells) and aiming at developing miRNA replacement therapy. Further studies with loss-of-function approaches would undoubtedly advance the understanding of the role of let-7-5p isoforms in PTGR under physiological conditions or particular circumstances. Nevertheless, in contrast to previous research that commonly investigates a single let-7-5p isoform in one study, this comparative study identifies the most promising let-7-5p isoforms against HCC cell viability, as well as regulating ABCC5 and LIN28B expression, which should inform the direction of future in vivo investigation of let-7 mono- and combination therapies.

In conclusion, this study established functional differences of 7 major let-7-5p isoforms in the regulation of target gene expression and HCC cell viability in vitro, whose effectiveness is dependent on the isoform’s sequence and abundance as well as interactions with specific targets and cellular microenvironment. Among them, let-7c-5p and let-7d-5p were revealed as effective regulators of efflux transporter ABCC5, indicating their potential for combination therapy as chemosensitizing agents to improve the treatment of HCC. Effects of BioRNA/let-7-5p isoforms against HCC cell viability were notably variable and not necessarily proportional to their regulatory strengths on a single oncogene, such as LIN28B. These findings highlight possible approaches to combat MDR in HCC and demonstrate the value of this novel class of miRNA molecules to investigate PTGR of ADME genes that are produced by innovative RNA bioengineering platform technology.

## Conflict of interest

The authors declare no competing interests.
