# Supplementary material for: Comparative effectiveness of 7 major human let-7-5p isoforms to modulate target gene expression in liver cells
Source: Drug Metab Dispos. 2026 Mar 2;54(4):100260. doi: 10.1016/j.dmd.2026.100260 (PMC13097275; doi:10.1016/j.dmd.2026.100260)

Supplementary Data

Comparative effectiveness of seven major human let-7-5p isoforms to modulate target gene expression in liver cells

Joseph M. Cronin, Mei-Juan Tu, Yimei Wang, and Ai-Ming Yu*

Department of Biochemistry and Molecular Medicine, University of California - Davis, School of Medicine, Sacramento, CA 95817, USA

Supplemental Table 1

Supplemental Figure 1

Supplemental Table 1. Sequences of hsa-let-7-5p isoforms and primers for stem-loop RT real-time qPCR analyses. Complementary interactions between hsa-let-7-5p isoforms and and corresponding stem-loop RT primers are underlined and color coded. Highlighted are corresponding hsa-let-7-5p segments and the isoform-specific qPCR primers. RT, reverse transcription; F, forward; R, reverse.

	**miRNA sequence (5' to 3')**
hsa-let-7a-5p	UGAGGUAGUAGGUUGUAUAGUU
hsa-let-7b-5p	UGAGGUAGUAGGUUGUGUGGUU
hsa-let-7c-5p	UGAGGUAGUAGGUUGUAUGGUU
hsa-let-7d-5p	AGAGGUAGUAGGUUGCAUAGUU
hsa-let-7e-5p	UGAGGUAGGAGGUUGUAUAGUU
hsa-let-7f-5p	UGAGGUAGUAGAUUGUAUAGUU
hsa-let-7g-5p	UGAGGUAGUAGUUUGUACAGUU
	**Stem-loop RT primer sequence (5' to 3')**
hsa-let-7a, d, e, f-5p	GTCGTATCCAGTGCAGGGTCCGAGGTATTCGCACTGGATACGACAACTAT
hsa-let-7b-5p	GTCGTATCCAGTGCAGGGTCCGAGGTATTCGCACTGGATACGACAACCAC
hsa-let-7c-5p	GTCGTATCCAGTGCAGGGTCCGAGGTATTCGCACTGGATACGACAACCAT
hsa-let-7g-5p	GTCGTATCCAGTGCAGGGTCCGAGGTATTCGCACTGGATACGACAACTGT
	**Real-time qPCR sequence (5' to 3')**
hsa-let-7a-5p F	CGCGCTGAGGTAGTAGGTTGT
hsa-let-7b-5p F	CGCGCATGAGGTAGTAGGTTGT
hsa-let-7c-5p F	CGCGCTGAGGTAGTAGGTTGT
hsa-let-7d-5p F	CGCGCAAGAGGTAGTAGGTTGC
hsa-let-7e-5p F	CGCGCATGAGGTAGGAGGTTGT
hsa-let-7f-5p F	GCCGCGTGAGGTAGTAGATTGT
hsa-let-7g-5p F	GCCGCGTGAGGTAGTAGTTTGT
Universal R	GTGCAGGGTCCGAGGT
U6 F	CTCGCTTCGGCAGCACA
U6 R	AACGCTTCACGAATTTGCGT

Supplemental Figure 1. Let-7-5p isoforms exhibit minimal to no regulation of (A) ABCC2/MRP2 and (B) ABCC4/MRP4 protein levels in human HCC cells. Huh7, HepG2, and Hep3B cells were treated with 15 nM of bioengineered let-7-5p or control RNA, or vehicle for 72 h, and Western blot analyses were performed to determine protein levels. Shown are representative blots. Protein band densities were normalized to corresponding total protein or β-actin levels, with control RNA group set as 1.0.

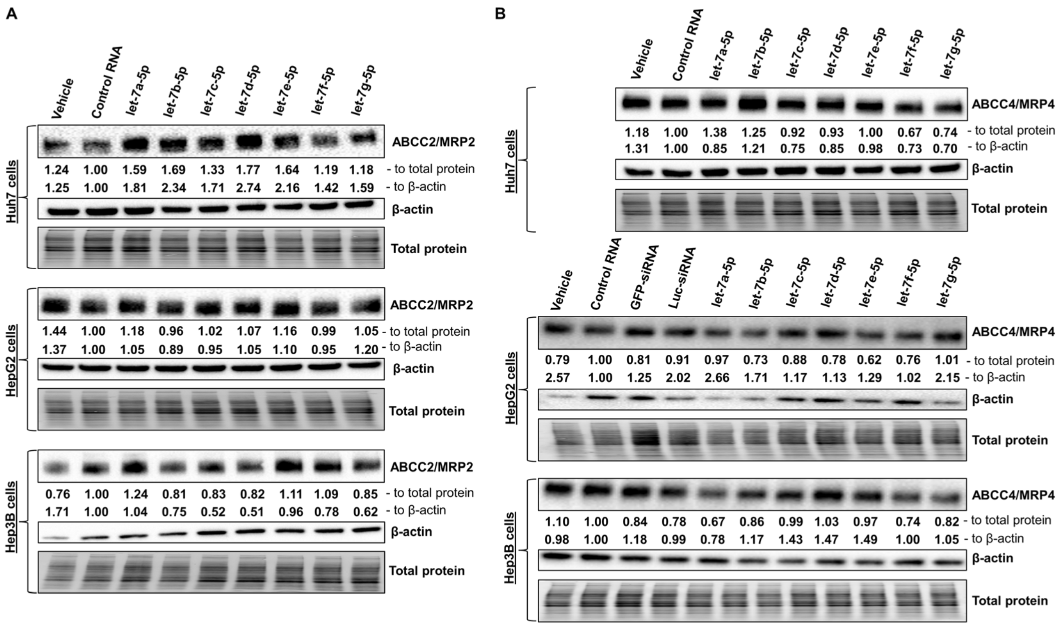

Supplement: Supplementary Table 1 and Supplementary Figure 1 [file mmc1.pdf]
